# Crystal structures of two cross-bridged chromium(III) tetra­aza­macrocycles

**DOI:** 10.1107/S1600536814019072

**Published:** 2014-08-30

**Authors:** Timothy J. Prior, Danny L. Maples, Randall D. Maples, Wesley A. Hoffert, Trenton H. Parsell, Jon D. Silversides, Stephen J. Archibald, Timothy J. Hubin

**Affiliations:** a Department of Chemistry, University of Hull, Cottingham Road, Hull HU6 7RX, England; b Department of Chemistry and Physics, Southwestern Oklahoma State University, Weatherford, OK 73096, USA; c Department of Natural Science, McPherson College, McPherson, KS 67460, USA

**Keywords:** crystal structure, chromium complexes, cross-bridged macrocycle

## Abstract

The crystal structure of di­chlorido­(4,10-dimethyl-1,4,7,10-tetra­aza­bicyclo­[5.5.2]tetra­deca­ne)chromium(III) hexa­fluorido­phosphate, [CrCl_2_(C_12_H_26_N_4_)]PF_6_, (I), has monoclinic symmetry (space group *P*2_1_/*n*) at 150 K. The structure of the related di­chlorido­(4,11-dimethyl-1,4,8,11-tetra­aza­bicyclo­[6.6.2]hexa­deca­ne)chromium(III) hexa­fluorido­phosphate, [CrCl_2_(C_14_H_30_N_4_)]PF_6_, (II), also displays monoclinic symmetry (space group *P*2_1_/*c*) at 150 K. In each case, the Cr^III^ ion is hexa­coordinate with two *cis* chloride ions and two non-adjacent N atoms bound *cis* equatorially and the other two non-adjacent N atoms bound *trans* axially in a *cis*-V conformation of the macrocycle. The extent of the distortion from the preferred octa­hedral coordination geometry of the Cr^III^ ion is determined by the parent macrocycle ring size, with the larger cross-bridged cyclam ring in (II) better able to accommodate this preference and the smaller cross-bridged cyclen ring in (I) requiring more distortion away from octa­hedral geometry.

## Abstract 2

Chromium(III) complexes of two ethyl­ene cross-bridged tetra­aza­macrocycles were prepared and structurally characterized in order to extend the coordination chemistry of this ligand type farther towards the early transition metals.

## Chemical context   

Ethyl­ene cross-bridged tetra­aza­macrocycles were introduced to coordination chemists in 1990 by Weisman and Wong (Weisman *et al.*, 1990 ▸). Since then, their transition metal complexes have become important to the fields of oxidation catalysis (Yin *et al.*, 2007 ▸; Dong *et al.*, 2013 ▸), medical/biological imaging (Boswell *et al.*, 2004 ▸; Sprague *et al.*, 2007 ▸; Silversides *et al.*, 2011 ▸) and chemokine receptor antagonism (Lewis *et al.*, 2005 ▸; Valks *et al.*, 2006 ▸; Smith *et al.*, 2012 ▸) due to the combination of restricted macrocycle configuration and kinetic inertness inherent to these ligands.Chromium(III) complexes have played an important role in characterizing new ligands due to their relative kinetic inertness (Cotton & Wilkinson, 1988 ▸). Yet, to date, only one report of the chromium coordination chemistry of these macrobicyclic ligands has appeared in the literature (Maples *et al.*, 2009 ▸). In order to expand the range of metal ions that can be coordinated by these remarkable ligands (Hubin, 2003 ▸), we are exploring further the structural chemistry of chromium cross-bridged tetra­aza­macrocyclic complexes and report synthesis and crystal structures of di­chlorido­(4,10-dimethyl-1,4,7,10-tetra­azabi­cyclo­[5.5.2]tetradeca­ne)chromium(III) hexa­fluorido­phos­phate, (I), and di­chlorido­(4,11-dimethyl-1,4,8,11-tetraazabi­cyclo­[6.6.2]hexadeca­ne)chromium(III)hexa­fluorido­phosphate, (II).[Chem scheme1] 
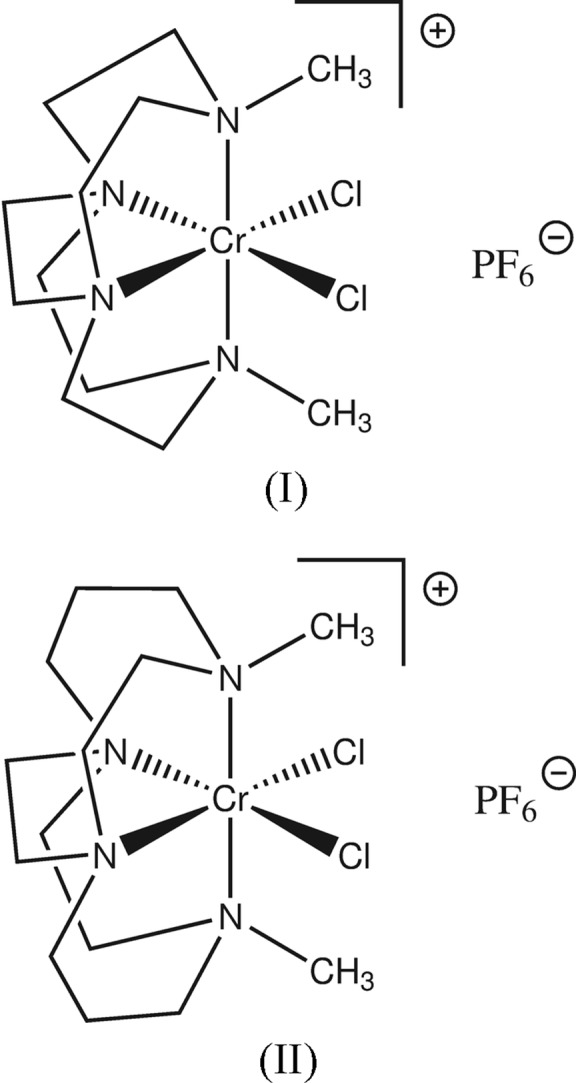


## Structural commentary   

**Figure 1:**
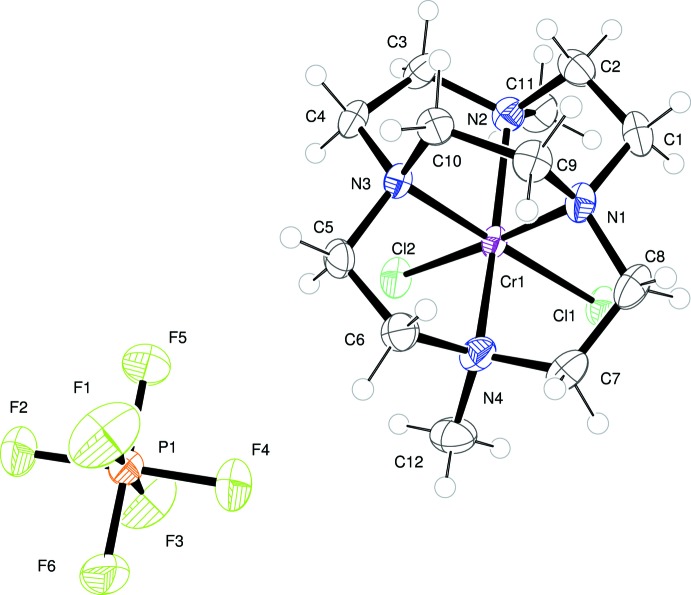
The mol­ecular entities of (I), with atoms shown as displacement ellipsoids at the 50% probability level.

Each of the title compounds crystallizes with a single positively-charged metal complex and one PF_6_^−^ anion in the asymmetric unit. The metal ion in each complex adopts a distorted octa­hedral geometry. The N atoms of each macrocycle occupy four coordination sites, while two chloride ions in a *cis* arrangement complete the coordination of Cr^III^. This so-called *cis*-V conformation, expected to be dictated by the ligand cross-bridge, is apparent for both of the complexes structurally characterized here. Figs. 1 ▸ and 2 ▸ illustrate the local geometry about Cr^III^ in (I) (dimethyl bridged-cyclen complex) and (II) (dimethyl bridged-cyclam complex), respectively. Apparently, neither the identity of the metal ion, nor that of the alkyl substituents affects this conformation. This same conformation has been seen in all known metal complexes with ethyl­ene cross-bridged cyclam and cyclen ligands.

The ring size of the parent macrocycle alters the degree to which the metal ion is engulfed by the bridged macrocycle. This is most clearly evident in the N2—Cr1—N4 bond angle between two axially bound nitro­gen atoms. This bond angle is 161.62 (11)° in the case of the smaller macrocycle, cylcen, while it is 171.44 (14)° for the cyclam complex. A larger bond angle, closer to linearity, indicates a better fit, or complementarity, between the ligand and the preferred octa­hedral geometry of the Cr^III^ ion. A more subtle difference in the N1—Cr1—N3 bond angles, *viz.* the equatorially bound N atoms, shows the same trend: this angle is 83.23 (10)° for the cyclen complex and 84.18 (13)° for the cyclam complex. Finally, the Cr—N bond lengths are somewhat affected by the ligand size as well. The mean of the four Cr—N bond lengths is 2.08 Å in (I), while this average is 2.12 Å in (II). The mean value for a number of Cr—N*R*_3_ bonds in the literature is 2.093 Å (σ = 0.044 Å) (Orpen *et al.*, 1989 ▸).

## Supra­molecular features   

There are no classical hydrogen bonds present in either (I) and (II) but each structure contains a great many C—H⋯F and C—H⋯Cl inter­actions which generate three-dimensional arrays. These inter­actions were identified from the standard criterion that the distance from the hydrogen atom to the hydrogen-bond acceptor should not exceed the sum of the radius of the acceptor plus 2 Å. Tables 1 ▸ and 2 ▸ contain full details of these inter­actions for (I) and (II), respectively.

**Figure 3:**
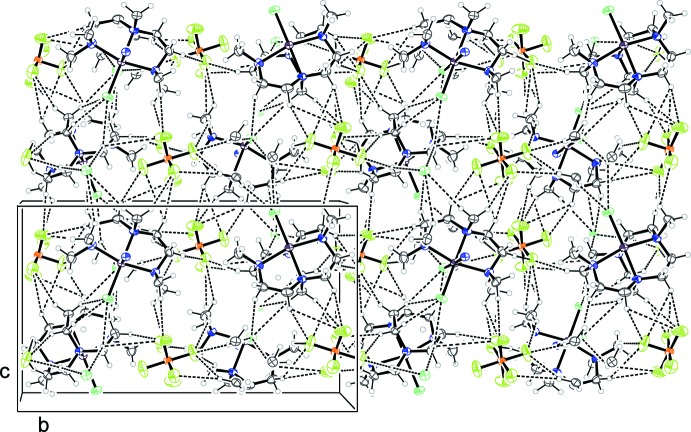
Crystal packing of (I), viewed perpendicular to the *bc* plane. Dashed lines represent halide⋯H—C inter­actions.

For (I), each PF_6_^−^ anion resides in a pocket between six metal complexes and there are C—H⋯F inter­actions to each of them. The mean C⋯F distance of those in Table 1 ▸ is 3.35 Å. Supplementary C—H⋯Cl intra­molecular contacts are present and inter­molecular inter­actions between neighbouring metal complexes are also observed. The overall effect of these inter­molecular inter­actions is to generate an extended network. One way to describe this is in terms of puckered sheets of the cationic complex and PF_6_^−^ anions that extend in the *bc* plane. Between these sheets further C—H⋯F and C—H⋯Cl inter­actions assemble these layers in an *ABAB* fashion along *a* to generate a densely packed three-dimensional array as shown in Fig. 3 ▸.

**Figure 4:**
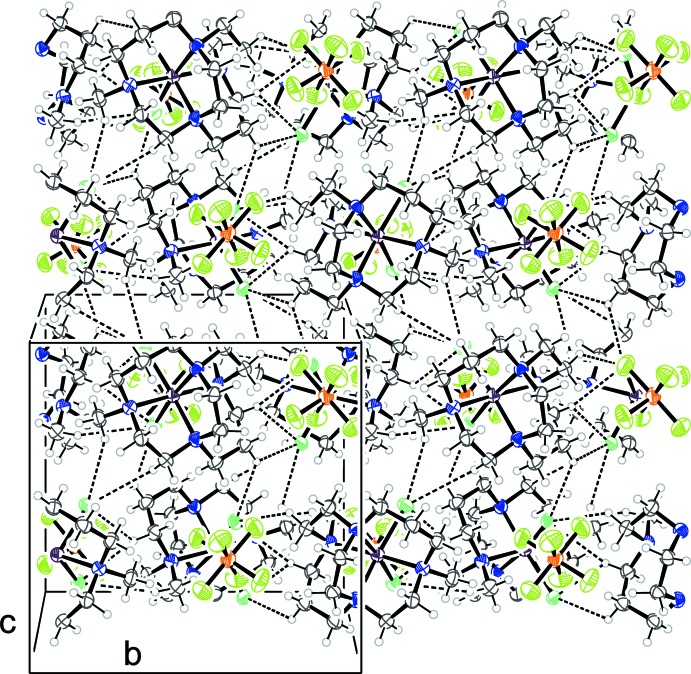
Crystal packing of (II), viewed perpendicular to the *bc* plane. Dashed lines represent halide⋯H—C inter­actions.

For (II), the arrangement is rather similar and again a three-dimensional array is constructed from nonclassical hydrogen bonds between the cations and anions. The PF_6_^−^ anion is located in a pocket formed from four metal complexes in a distorted tetra­hedral arrangement and forms C—H⋯F inter­actions to each of them, with a mean C⋯F distance of 3.23 Å. Further C—H⋯Cl inter­actions are also present. In a similar fashion to (I), these nonclassical inter­actions assemble the cations and anions into puckered sheets that extend in the *bc* plane. The sheets are then *ABAB* stacked along *a* as shown in Fig. 4 ▸.

## Database survey   

The structures of three complexes that are directly analogous to (I) have been reported. These are the manganese (Hubin *et al.*, 2001 ▸), iron (McClain *et al.*, 2006 ▸) and cobalt (Hubin *et al.*, 2002 ▸) analogues. Each of these contains the metal in the trivalent state. For the Mn and Fe examples, the geometry about the metal is similar to that for Cr, but the bond angles formed by the two axially bound N atoms are smaller [155.01 (11) and 153.20 (9) °, respectively]. Similarly, the bite angles of the two equatorially bound N atoms are also noticeably smaller; these are 81.29 (11) and 78.62 (8)° for Mn and Fe analogues, respectively. However, the mean *M*—N bond length is longer for Mn and Fe examples: 2.153 and 2.167 Å, respectively. These differences in geometry reflect the smaller size of the Mn^III^ and Fe^III^ ions, and the possibility of a Jahn–Teller distortion for Mn^III^, but the greater ligand field stabilization energy (LFSE) for Cr^III^ yields shorter bond lengths. The Co^III^ analogue is rather different because it is in a low spin state. The axial N—Co—N bond angle is 168.8 (4)° and the equatorial bond angle is 87.2 (4)°. As expected, the mean bond length is shorter for the Co case at 1.978 Å. The smaller, low-spin Co^III^ ion fits into the pocket of the macrocyle better than Cr^III^.

Chromium(III) complexes similar to (I) and (II) but crystallized with different anions have been reported before (Maples *et al.*, 2009 ▸). The chloride analogue of (I) has bond angles of 160.83 (19) and 83.50 (18)° about the chromium ion and a mean Cr—N bond length of 2.08 Å, which are in good agreement with (I), demonstrating the counter-anion has very little effect on the coordination about the metal. A cyclen-based macrocycle with benzyl groups replacing the methyl groups in (I), has key bond angles 160.35 (19) and 83.6 (2)° and a mean Cr—N bond length of 2.09 Å (Maples *et al.*, 2009 ▸). The pocket in the macrocycle is of a similar shape in this example but slightly enlarged because of the pendant benzyl groups.

The chloride analogue of (II) (Maples *et al.*, 2009 ▸) displays a similarly sized pocket; the N—Cr—N axial bond angle is 172.46 (11)° and the equatorial angle is 84.63 (11)°, while mean Cr—N bond length is 2.12 Å. In line with the observation in (I) and (II), the pocket of the cyclam-derived ligand is better able to accomodate the octa­hedrally surrounded Cr^III^ ion and displays larger bond lengths than the cyclen equivalent.

## Synthesis and crystallization   

The cross-bridged ligands were prepared according to literature procedures (Weisman *et al.*, 1990 ▸; Wong *et al.*, 2000 ▸). The title complexes were prepared by a procedure slightly modified from those found in Hubin *et al.* (2001 ▸) for other trivalent metal ions. In an inert atmosphere glove-box, 1 mmol of the respective ligand was dissolved in 20 ml of anhydrous di­methyl­formamide in a 50 ml Erlenmeyer flask. 1 mmol of anhydrous chromium(II) chloride was added to the stirring ligand solution. The reaction was allowed to stir at room temperature overnight. The solution was then filtered through filter paper and the solvent was removed under vacuum to give blue–violet solids. In the glove-box, this divalent complex was dissolved in 20 ml of methanol in a round-bottomed flask. Five equivalents of NH_4_PF_6_ (5 mmol, 0.815 g) were dissolved in the solution. The flask was removed from the glove-box with a stopper to protect it from air. In a fume hood, a stream of nitro­gen gas was directed over the surface of the solution. Four to six drops of Br_2_ were added and the reaction was stirred for 15 min. Bright purple precipitates formed immediately. The nitro­gen gas was then allowed to bubble through the solution for 15 min to remove excess Br_2_. The flask was then stoppered and placed in a freezer for 30 min to complete the precipitation. The purple solid product was collected by vacuum filtration on a glass frit and washed with methanol and then ether. Crystals suitable for X-ray diffraction (purple blocks) were grown from the slow evaporation of aqueous solutions of the product.

## Refinement   

Crystal data, data collection and structure refinement details are summarized in Table 3 ▸. H atoms were placed in idealised positions and refined using a riding model, with C—H = 0.98 and 0.99 Å for –CH_3_ and –CH_2_– groups, respectively, and with *U*_iso_(H) values of, respectively, 1.5 and 1.2 times *U*_eq_ of the carrier atom.

In (I), there is evidence for a very small degree of disorder (10%) in the position of the PF_6_^−^ anions. Refinement with a second orientation for this anion did not lead to a substantial improve in the fit. A model with a single orientation was therefore retained.

The structure of (II) is presented in *P*2_1_/*n*, consistent with manganese and cobalt analogues (Hubin *et al.*, 2001 ▸, 2002 ▸), rather than the *P*2_1_/*c* setting of the iron analogue (McClain *et al.*, 2006 ▸) which has β ≃ 120°.

## Supplementary Material

Crystal structure: contains datablock(s) I, II. DOI: 10.1107/S1600536814019072/wm5051sup1.cif

Structure factors: contains datablock(s) I. DOI: 10.1107/S1600536814019072/wm5051Isup2.hkl

Structure factors: contains datablock(s) II. DOI: 10.1107/S1600536814019072/wm5051IIsup3.hkl

CCDC references: 1020729, 1020730

Additional supporting information:  crystallographic information; 3D view; checkCIF report

## Figures and Tables

**Figure 2:**
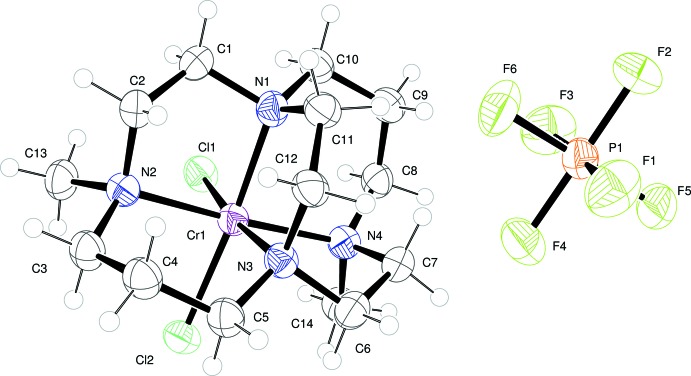
The mol­ecular entities of (II), with atoms shown as displacement ellipsoids at the 50% probability level.

**Table 1:** Hydrogen-bond geometry (Å, °) for (I)

*D*—H⋯*A*	*D*—H	H⋯*A*	*D*⋯*A*	*D*—H⋯*A*
C1—H1*A*⋯F6^i^	0.99	2.53	3.347 (4)	140
C1—H1*B*⋯F3^ii^	0.99	2.46	3.410 (5)	162
C2—H2*A*⋯F4^i^	0.99	2.48	3.407 (4)	155
C3—H3*A*⋯F1^iii^	0.99	2.51	3.500 (4)	177
C4—H4*A*⋯F6^iv^	0.99	2.36	3.175 (4)	139
C6—H6*B*⋯F4	0.99	2.30	3.238 (4)	158
C1—H1*A*⋯Cl1	0.99	2.82	3.393 (4)	118
C4—H4*B*⋯Cl2	0.99	2.63	3.137 (3)	112
C5—H5*A*⋯Cl2	0.99	2.74	3.324 (3)	118
C6—H6*A*⋯Cl2^v^	0.99	2.77	3.447 (3)	126
C8—H8*B*⋯Cl1	0.99	2.73	3.203 (4)	110
C12—H12*B*⋯Cl2	0.98	2.78	3.329 (4)	116

Symmetry codes: (i) 
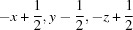
; (ii) 
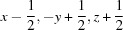
; (iii) 
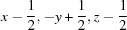
; (iv) 
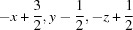
; (v) 
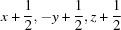
.

**Table 2:** Hydrogen-bond geometry (Å, °) for (II)

*D*—H⋯*A*	*D*—H	H⋯*A*	*D*⋯*A*	*D*—H⋯*A*
C1—H1*B*⋯F5^i^	0.99	2.41	3.318 (5)	153
C2—H2*A*⋯F1^ii^	0.99	2.50	3.094 (5)	119
C2—H2*A*⋯F6^ii^	0.99	2.53	3.223 (6)	127
C7—H7*A*⋯F4	0.99	2.55	3.098 (6)	115
C12—H12*B*⋯F2^ii^	0.99	2.51	3.393 (6)	149
C3—H3*A*⋯Cl2	0.99	2.73	3.280 (5)	115
C5—H5*B*⋯Cl2	0.99	2.70	3.287 (5)	119
C8—H8*A*⋯Cl1	0.99	2.70	3.258 (5)	116
C8—H8*B*⋯Cl1^iii^	0.99	2.82	3.778 (4)	162
C10—H10*B*⋯Cl1	0.99	2.70	3.270 (5)	117
C13—H13*A*⋯Cl1	0.98	2.68	3.157 (5)	111
C13—H13*A*⋯Cl2^iv^	0.98	2.73	3.572 (4)	144
C14—H14*B*⋯Cl2	0.98	2.69	3.141 (5)	108

Symmetry codes: (i) 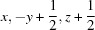; (ii) 
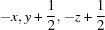
; (iii) 
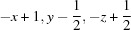
; (iv) 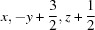.

**Table 3:** Experimental details

	(I)	(II)
Crystal data		
Chemical formula	[CrCl_2_(C_12_H_26_N_4_)]PF_6_	[CrCl_2_(C_14_H_30_N_4_)]PF_6_
*M*_r_	494.24	522.29
Crystal system, space group	Monoclinic, *P*2_1_/*n*	Monoclinic, *P*2_1_/*c*
Temperature (K)	150	150
*a*, *b*, *c* (Å)	8.2271 (10), 19.957 (2), 12.0474 (17)	13.6801 (19), 12.437 (2), 12.3864 (17)
β (°)	96.374 (11)	102.028 (11)
*V* (Å^3^)	1965.8 (4)	2061.1 (5)
*Z*	4	4
Radiation type	Mo *K*α	Mo *K*α
μ (mm^−1^)	1.00	0.95
Crystal size (mm)	0.10 × 0.10 × 0.08	0.15 × 0.15 × 0.06

Data collection		
Diffractometer	Stoe IPDS2	Stoe IPDS2
Absorption correction	Analytical [a face-indexed absorption correction was applied; *X-AREA* (Stoe & Cie, 2002 ▸)]	Analytical [a face-indexed absorption correction was applied; *X-AREA* (Stoe & Cie, 2002 ▸)]
*T*_min_, *T*_max_	0.827, 0.915	0.778, 0.901
No. of measured, independent and observed [*I* > 2σ(*I*)] reflections	22999, 4501, 2798	13450, 4158, 2073
*R*_int_	0.091	0.091
(sin θ/λ)_max_ (Å^−1^)	0.650	0.622

Refinement		
*R*[*F* ^2^ > 2σ(*F* ^2^)], *wR*(*F* ^2^), *S*	0.041, 0.096, 0.88	0.045, 0.109, 0.78
No. of reflections	4501	4158
No. of parameters	235	255
H-atom treatment	H-atom parameters constrained	H-atom parameters constrained
Δρ_max_, Δρ_min_ (e Å^−3^)	0.77, −0.49	0.33, −0.79

Computer programs: *X-AREA* (Stoe & Cie, 2002 ▸), *SHELXS86*, *SHELXL2013* and *SHELXTL* (Sheldrick, 2008 ▸).

## supplementary crystallographic information

### (I) Dichlorido(4,10-dimethyl-1,4,7,10-tetraazabicyclo[5.5.2]tetradecane)chromium(III) hexafluoridophosphate Crystal data

**Table:**

[CrCl_2_(C_12_H_26_N_4_)]PF_6_	*F*(000) = 1012
*M**_r_* = 494.24	*D*_x_ = 1.670 Mg m^−^^3^
Monoclinic, *P*2_1_/*n*	Mo *K*α radiation, λ = 0.71073 Å
*a* = 8.2271 (10) Å	Cell parameters from 12040 reflections
*b* = 19.957 (2) Å	θ = 2.7–31.2°
*c* = 12.0474 (17) Å	µ = 1.00 mm^−^^1^
β = 96.374 (11)°	*T* = 150 K
*V* = 1965.8 (4) Å^3^	Block, purple
*Z* = 4	0.10 × 0.10 × 0.08 mm

### (I) Dichlorido(4,10-dimethyl-1,4,7,10-tetraazabicyclo[5.5.2]tetradecane)chromium(III) hexafluoridophosphate Data collection

**Table:**

Stoe IPDS2 diffractometer	4501 independent reflections
Radiation source: fine-focus sealed tube	2798 reflections with *I* > 2σ(*I*)
Detector resolution: 6.67 pixels mm^-1^	*R*_int_ = 0.091
ω–scans	θ_max_ = 27.5°, θ_min_ = 2.7°
Absorption correction: analytical [a face-indexed absorption correction was applied; *X-AREA* (Stoe & Cie, 2002)]	*h* = −10→10
*T*_min_ = 0.827, *T*_max_ = 0.915	*k* = −25→25
22999 measured reflections	*l* = −14→15

### (I) Dichlorido(4,10-dimethyl-1,4,7,10-tetraazabicyclo[5.5.2]tetradecane)chromium(III) hexafluoridophosphate Refinement

**Table:**

Refinement on *F*^2^	0 restraints
Least-squares matrix: full	Hydrogen site location: inferred from neighbouring sites
*R*[*F*^2^ > 2σ(*F*^2^)] = 0.041	H-atom parameters constrained
*wR*(*F*^2^) = 0.096	*w* = 1/[σ^2^(*F*_o_^2^) + (0.0474*P*)^2^] where *P* = (*F*_o_^2^ + 2*F*_c_^2^)/3
*S* = 0.88	(Δ/σ)_max_ < 0.001
4501 reflections	Δρ_max_ = 0.77 e Å^−^^3^
235 parameters	Δρ_min_ = −0.49 e Å^−^^3^

### (I) Dichlorido(4,10-dimethyl-1,4,7,10-tetraazabicyclo[5.5.2]tetradecane)chromium(III) hexafluoridophosphate Special details

**Table:**

Geometry. All e.s.d.'s (except the e.s.d. in the dihedral angle between two l.s. planes) are estimated using the full covariance matrix. The cell e.s.d.'s are taken into account individually in the estimation of e.s.d.'s in distances, angles and torsion angles; correlations between e.s.d.'s in cell parameters are only used when they are defined by crystal symmetry. An approximate (isotropic) treatment of cell e.s.d.'s is used for estimating e.s.d.'s involving l.s. planes.

### (I) Dichlorido(4,10-dimethyl-1,4,7,10-tetraazabicyclo[5.5.2]tetradecane)chromium(III) hexafluoridophosphate Fractional atomic coordinates and isotropic or equivalent isotropic displacement parameters (Å^2^)

**Table:**

	*x*	*y*	*z*	*U*_iso_*/*U*_eq_	
Cr1	0.21553 (5)	0.19169 (2)	0.20979 (4)	0.02173 (12)	
Cl1	−0.06600 (7)	0.20275 (4)	0.17145 (7)	0.03159 (19)	
Cl2	0.25268 (8)	0.23403 (4)	0.03552 (7)	0.03325 (19)	
N1	0.2035 (3)	0.15406 (14)	0.3675 (2)	0.0274 (6)	
N2	0.2399 (3)	0.09097 (13)	0.1641 (2)	0.0276 (6)	
N3	0.4628 (3)	0.18082 (13)	0.2517 (2)	0.0234 (5)	
N4	0.2408 (3)	0.28159 (14)	0.2997 (2)	0.0296 (6)	
C1	0.1252 (4)	0.08715 (17)	0.3466 (3)	0.0348 (8)	
H1A	0.0099	0.0927	0.3144	0.042*	
H1B	0.1267	0.0621	0.4177	0.042*	
C2	0.2193 (4)	0.04929 (17)	0.2664 (3)	0.0340 (7)	
H2A	0.1606	0.0074	0.2435	0.041*	
H2B	0.3283	0.0371	0.3042	0.041*	
C3	0.4087 (3)	0.08276 (17)	0.1267 (3)	0.0328 (8)	
H3A	0.3976	0.0776	0.0445	0.039*	
H3B	0.4586	0.0412	0.1599	0.039*	
C4	0.5235 (3)	0.14187 (17)	0.1595 (3)	0.0300 (7)	
H4A	0.6348	0.1249	0.1837	0.036*	
H4B	0.5296	0.1714	0.0940	0.036*	
C5	0.5233 (3)	0.25153 (16)	0.2599 (3)	0.0298 (7)	
H5A	0.5118	0.2726	0.1850	0.036*	
H5B	0.6402	0.2524	0.2899	0.036*	
C6	0.4221 (3)	0.28971 (17)	0.3373 (3)	0.0325 (8)	
H6A	0.4467	0.2726	0.4145	0.039*	
H6B	0.4515	0.3378	0.3372	0.039*	
C7	0.1464 (4)	0.27476 (19)	0.3992 (3)	0.0370 (8)	
H7A	0.0446	0.3014	0.3858	0.044*	
H7B	0.2124	0.2937	0.4655	0.044*	
C8	0.1016 (4)	0.20179 (19)	0.4244 (3)	0.0379 (8)	
H8A	0.1186	0.1940	0.5061	0.045*	
H8B	−0.0154	0.1939	0.3988	0.045*	
C9	0.3681 (3)	0.14731 (18)	0.4344 (3)	0.0321 (7)	
H9A	0.3697	0.1062	0.4805	0.038*	
H9B	0.3867	0.1861	0.4854	0.038*	
C10	0.5051 (3)	0.14384 (16)	0.3594 (3)	0.0275 (7)	
H10A	0.6059	0.1633	0.3993	0.033*	
H10B	0.5276	0.0963	0.3429	0.033*	
C11	0.1158 (4)	0.06839 (19)	0.0721 (3)	0.0377 (8)	
H11A	0.0059	0.0742	0.0947	0.057*	
H11B	0.1338	0.0210	0.0559	0.057*	
H11C	0.1262	0.0951	0.0050	0.057*	
C12	0.1830 (4)	0.34219 (19)	0.2353 (4)	0.0428 (9)	
H12A	0.0660	0.3378	0.2103	0.064*	
H12B	0.2437	0.3469	0.1701	0.064*	
H12C	0.2012	0.3819	0.2829	0.064*	
P1	0.74031 (9)	0.44740 (4)	0.22662 (7)	0.0291 (2)	
F1	0.8570 (3)	0.43093 (16)	0.3353 (2)	0.0756 (8)	
F2	0.8987 (2)	0.46269 (12)	0.1662 (2)	0.0570 (6)	
F3	0.6248 (3)	0.46395 (16)	0.1167 (2)	0.0744 (8)	
F4	0.5828 (3)	0.43324 (12)	0.2878 (2)	0.0604 (7)	
F5	0.7476 (3)	0.37224 (11)	0.1869 (3)	0.0675 (8)	
F6	0.7324 (3)	0.52314 (12)	0.2644 (3)	0.0639 (7)	

### (I) Dichlorido(4,10-dimethyl-1,4,7,10-tetraazabicyclo[5.5.2]tetradecane)chromium(III) hexafluoridophosphate Atomic displacement parameters (Å^2^)

**Table:**

	*U*^11^	*U*^22^	*U*^33^	*U*^12^	*U*^13^	*U*^23^
Cr1	0.01590 (19)	0.0277 (3)	0.0219 (3)	−0.00047 (18)	0.00316 (17)	0.0011 (2)
Cl1	0.0156 (3)	0.0430 (5)	0.0361 (5)	0.0006 (3)	0.0023 (3)	0.0020 (4)
Cl2	0.0254 (3)	0.0468 (5)	0.0273 (4)	−0.0002 (3)	0.0018 (3)	0.0092 (4)
N1	0.0232 (11)	0.0368 (16)	0.0228 (14)	−0.0009 (10)	0.0049 (10)	0.0025 (12)
N2	0.0241 (11)	0.0302 (15)	0.0279 (15)	−0.0014 (10)	0.0011 (10)	−0.0035 (12)
N3	0.0173 (10)	0.0293 (14)	0.0237 (13)	−0.0008 (9)	0.0030 (9)	−0.0003 (11)
N4	0.0233 (11)	0.0322 (15)	0.0331 (16)	0.0037 (10)	0.0024 (11)	−0.0033 (12)
C1	0.0297 (15)	0.042 (2)	0.0334 (19)	−0.0101 (14)	0.0055 (14)	0.0085 (16)
C2	0.0342 (15)	0.0284 (18)	0.038 (2)	−0.0054 (13)	−0.0005 (14)	0.0049 (16)
C3	0.0265 (14)	0.0368 (19)	0.036 (2)	0.0060 (13)	0.0067 (13)	−0.0101 (16)
C4	0.0200 (13)	0.0403 (19)	0.0306 (18)	0.0043 (12)	0.0073 (12)	0.0006 (15)
C5	0.0197 (12)	0.0329 (18)	0.0364 (19)	−0.0056 (12)	0.0014 (12)	0.0017 (15)
C6	0.0244 (14)	0.0320 (19)	0.040 (2)	−0.0022 (12)	−0.0011 (13)	−0.0051 (15)
C7	0.0319 (15)	0.047 (2)	0.0324 (19)	0.0083 (15)	0.0065 (14)	−0.0095 (17)
C8	0.0336 (15)	0.052 (2)	0.0302 (18)	0.0077 (15)	0.0137 (14)	−0.0011 (17)
C9	0.0321 (15)	0.0389 (19)	0.0248 (17)	0.0005 (14)	0.0011 (13)	0.0016 (15)
C10	0.0249 (13)	0.0317 (17)	0.0244 (17)	0.0008 (12)	−0.0035 (12)	0.0032 (14)
C11	0.0313 (15)	0.045 (2)	0.035 (2)	−0.0027 (14)	−0.0049 (14)	−0.0101 (17)
C12	0.0393 (17)	0.034 (2)	0.053 (3)	0.0085 (15)	−0.0046 (16)	0.0024 (18)
P1	0.0267 (4)	0.0287 (4)	0.0326 (5)	0.0003 (3)	0.0059 (3)	−0.0026 (4)
F1	0.0721 (16)	0.109 (2)	0.0429 (15)	0.0339 (15)	−0.0032 (13)	0.0063 (16)
F2	0.0442 (11)	0.0542 (14)	0.0786 (18)	−0.0083 (10)	0.0329 (11)	−0.0070 (13)
F3	0.0674 (15)	0.107 (2)	0.0452 (15)	0.0192 (15)	−0.0114 (12)	0.0022 (15)
F4	0.0486 (12)	0.0574 (15)	0.0815 (19)	−0.0082 (10)	0.0357 (12)	−0.0007 (13)
F5	0.0616 (14)	0.0366 (13)	0.109 (2)	−0.0068 (10)	0.0292 (14)	−0.0234 (14)
F6	0.0456 (12)	0.0412 (14)	0.107 (2)	−0.0014 (10)	0.0159 (12)	−0.0269 (14)

### (I) Dichlorido(4,10-dimethyl-1,4,7,10-tetraazabicyclo[5.5.2]tetradecane)chromium(III) hexafluoridophosphate Geometric parameters (Å, º)

**Table:**

Cr1—N3	2.053 (2)	C5—C6	1.522 (4)
Cr1—N1	2.056 (3)	C5—H5A	0.9900
Cr1—N4	2.094 (3)	C5—H5B	0.9900
Cr1—N2	2.100 (3)	C6—H6A	0.9900
Cr1—Cl2	2.3147 (9)	C6—H6B	0.9900
Cr1—Cl1	2.3215 (8)	C7—C8	1.541 (5)
N1—C8	1.486 (4)	C7—H7A	0.9900
N1—C1	1.492 (4)	C7—H7B	0.9900
N1—C9	1.503 (4)	C8—H8A	0.9900
N2—C11	1.491 (4)	C8—H8B	0.9900
N2—C2	1.512 (4)	C9—C10	1.523 (4)
N2—C3	1.516 (3)	C9—H9A	0.9900
N3—C4	1.486 (4)	C9—H9B	0.9900
N3—C5	1.496 (4)	C10—H10A	0.9900
N3—C10	1.499 (4)	C10—H10B	0.9900
N4—C12	1.486 (5)	C11—H11A	0.9800
N4—C7	1.505 (4)	C11—H11B	0.9800
N4—C6	1.519 (4)	C11—H11C	0.9800
C1—C2	1.507 (5)	C12—H12A	0.9800
C1—H1A	0.9900	C12—H12B	0.9800
C1—H1B	0.9900	C12—H12C	0.9800
C2—H2A	0.9900	P1—F1	1.570 (3)
C2—H2B	0.9900	P1—F3	1.577 (3)
C3—C4	1.535 (5)	P1—F5	1.578 (2)
C3—H3A	0.9900	P1—F6	1.582 (2)
C3—H3B	0.9900	P1—F4	1.585 (2)
C4—H4A	0.9900	P1—F2	1.591 (2)
C4—H4B	0.9900		
			
N3—Cr1—N1	83.23 (10)	N3—C5—C6	108.2 (2)
N3—Cr1—N4	85.68 (10)	N3—C5—H5A	110.1
N1—Cr1—N4	81.21 (11)	C6—C5—H5A	110.1
N3—Cr1—N2	80.93 (9)	N3—C5—H5B	110.1
N1—Cr1—N2	84.74 (11)	C6—C5—H5B	110.1
N4—Cr1—N2	161.62 (11)	H5A—C5—H5B	108.4
N3—Cr1—Cl2	91.97 (7)	N4—C6—C5	110.5 (3)
N1—Cr1—Cl2	175.19 (7)	N4—C6—H6A	109.6
N4—Cr1—Cl2	98.09 (8)	C5—C6—H6A	109.6
N2—Cr1—Cl2	94.91 (8)	N4—C6—H6B	109.6
N3—Cr1—Cl1	177.18 (8)	C5—C6—H6B	109.6
N1—Cr1—Cl1	93.99 (7)	H6A—C6—H6B	108.1
N4—Cr1—Cl1	93.50 (7)	N4—C7—C8	113.5 (3)
N2—Cr1—Cl1	99.27 (7)	N4—C7—H7A	108.9
Cl2—Cr1—Cl1	90.81 (3)	C8—C7—H7A	108.9
C8—N1—C1	113.3 (2)	N4—C7—H7B	108.9
C8—N1—C9	109.3 (3)	C8—C7—H7B	108.9
C1—N1—C9	110.9 (3)	H7A—C7—H7B	107.7
C8—N1—Cr1	106.2 (2)	N1—C8—C7	110.8 (2)
C1—N1—Cr1	103.6 (2)	N1—C8—H8A	109.5
C9—N1—Cr1	113.31 (17)	C7—C8—H8A	109.5
C11—N2—C2	108.1 (2)	N1—C8—H8B	109.5
C11—N2—C3	108.7 (2)	C7—C8—H8B	109.5
C2—N2—C3	111.8 (2)	H8A—C8—H8B	108.1
C11—N2—Cr1	114.0 (2)	N1—C9—C10	111.6 (3)
C2—N2—Cr1	106.88 (19)	N1—C9—H9A	109.3
C3—N2—Cr1	107.38 (18)	C10—C9—H9A	109.3
C4—N3—C5	114.0 (2)	N1—C9—H9B	109.3
C4—N3—C10	108.9 (2)	C10—C9—H9B	109.3
C5—N3—C10	111.3 (2)	H9A—C9—H9B	108.0
C4—N3—Cr1	106.11 (18)	N3—C10—C9	112.0 (2)
C5—N3—Cr1	103.27 (17)	N3—C10—H10A	109.2
C10—N3—Cr1	113.16 (16)	C9—C10—H10A	109.2
C12—N4—C7	109.0 (3)	N3—C10—H10B	109.2
C12—N4—C6	108.4 (2)	C9—C10—H10B	109.2
C7—N4—C6	110.4 (3)	H10A—C10—H10B	107.9
C12—N4—Cr1	114.8 (2)	N2—C11—H11A	109.5
C7—N4—Cr1	107.7 (2)	N2—C11—H11B	109.5
C6—N4—Cr1	106.47 (18)	H11A—C11—H11B	109.5
N1—C1—C2	108.3 (2)	N2—C11—H11C	109.5
N1—C1—H1A	110.0	H11A—C11—H11C	109.5
C2—C1—H1A	110.0	H11B—C11—H11C	109.5
N1—C1—H1B	110.0	N4—C12—H12A	109.5
C2—C1—H1B	110.0	N4—C12—H12B	109.5
H1A—C1—H1B	108.4	H12A—C12—H12B	109.5
C1—C2—N2	111.0 (3)	N4—C12—H12C	109.5
C1—C2—H2A	109.4	H12A—C12—H12C	109.5
N2—C2—H2A	109.4	H12B—C12—H12C	109.5
C1—C2—H2B	109.4	F1—P1—F3	179.36 (15)
N2—C2—H2B	109.4	F1—P1—F5	90.78 (16)
H2A—C2—H2B	108.0	F3—P1—F5	89.08 (17)
N2—C3—C4	113.5 (2)	F1—P1—F6	90.07 (16)
N2—C3—H3A	108.9	F3—P1—F6	90.06 (16)
C4—C3—H3A	108.9	F5—P1—F6	179.05 (17)
N2—C3—H3B	108.9	F1—P1—F4	91.76 (15)
C4—C3—H3B	108.9	F3—P1—F4	88.88 (15)
H3A—C3—H3B	107.7	F5—P1—F4	91.85 (13)
N3—C4—C3	110.3 (2)	F6—P1—F4	88.55 (13)
N3—C4—H4A	109.6	F1—P1—F2	88.05 (14)
C3—C4—H4A	109.6	F3—P1—F2	91.32 (15)
N3—C4—H4B	109.6	F5—P1—F2	89.00 (12)
C3—C4—H4B	109.6	F6—P1—F2	90.60 (13)
H4A—C4—H4B	108.1	F4—P1—F2	179.13 (14)
			
C8—N1—C1—C2	167.0 (3)	C7—N4—C6—C5	141.4 (3)
C9—N1—C1—C2	−69.6 (3)	Cr1—N4—C6—C5	24.8 (3)
Cr1—N1—C1—C2	52.3 (3)	N3—C5—C6—N4	−52.8 (3)
N1—C1—C2—N2	−51.4 (3)	C12—N4—C7—C8	140.9 (3)
C11—N2—C2—C1	−100.3 (3)	C6—N4—C7—C8	−100.1 (3)
C3—N2—C2—C1	140.1 (3)	Cr1—N4—C7—C8	15.7 (3)
Cr1—N2—C2—C1	22.8 (3)	C1—N1—C8—C7	−155.4 (3)
C11—N2—C3—C4	138.1 (3)	C9—N1—C8—C7	80.4 (3)
C2—N2—C3—C4	−102.6 (3)	Cr1—N1—C8—C7	−42.3 (3)
Cr1—N2—C3—C4	14.3 (3)	N4—C7—C8—N1	17.3 (4)
C5—N3—C4—C3	−157.4 (3)	C8—N1—C9—C10	−140.2 (3)
C10—N3—C4—C3	77.6 (3)	C1—N1—C9—C10	94.1 (3)
Cr1—N3—C4—C3	−44.5 (3)	Cr1—N1—C9—C10	−21.9 (3)
N2—C3—C4—N3	19.5 (4)	C4—N3—C10—C9	−140.6 (3)
C4—N3—C5—C6	166.7 (3)	C5—N3—C10—C9	92.9 (3)
C10—N3—C5—C6	−69.7 (3)	Cr1—N3—C10—C9	−22.9 (3)
Cr1—N3—C5—C6	52.0 (3)	N1—C9—C10—N3	28.9 (4)
C12—N4—C6—C5	−99.2 (3)		

### (I) Dichlorido(4,10-dimethyl-1,4,7,10-tetraazabicyclo[5.5.2]tetradecane)chromium(III) hexafluoridophosphate Hydrogen-bond geometry (Å, º)

**Table:**

*D*—H···*A*	*D*—H	H···*A*	*D*···*A*	*D*—H···*A*
C1—H1*A*···F6^i^	0.99	2.53	3.347 (4)	140
C1—H1*B*···F3^ii^	0.99	2.46	3.410 (5)	162
C2—H2*A*···F4^i^	0.99	2.48	3.407 (4)	155
C3—H3*A*···F1^iii^	0.99	2.51	3.500 (4)	177
C4—H4*A*···F6^iv^	0.99	2.36	3.175 (4)	139
C6—H6*B*···F4	0.99	2.30	3.238 (4)	158
C1—H1*A*···Cl1	0.99	2.82	3.393 (4)	118
C4—H4*B*···Cl2	0.99	2.63	3.137 (3)	112
C5—H5*A*···Cl2	0.99	2.74	3.324 (3)	118
C6—H6*A*···Cl2^v^	0.99	2.77	3.447 (3)	126
C8—H8*B*···Cl1	0.99	2.73	3.203 (4)	110
C12—H12*B*···Cl2	0.98	2.78	3.329 (4)	116

Symmetry codes: (i) −*x*+1/2, *y*−1/2, −*z*+1/2; (ii) *x*−1/2, −*y*+1/2, *z*+1/2; (iii) *x*−1/2, −*y*+1/2, *z*−1/2; (iv) −*x*+3/2, *y*−1/2, −*z*+1/2; (v) *x*+1/2, −*y*+1/2, *z*+1/2.

### (II) Dichlorido(4,11-dimethyl-1,4,8,11-tetraazabicyclo[6.6.2]hexadecane)chromium(III) hexafluorophosphate Crystal data

**Table:**

[CrCl_2_(C_14_H_30_N_4_)]PF_6_	*F*(000) = 1076
*M**_r_* = 522.29	*D*_x_ = 1.683 Mg m^−^^3^
Monoclinic, *P*2_1_/*c*	Mo *K*α radiation, λ = 0.71073 Å
*a* = 13.6801 (19) Å	Cell parameters from 9091 reflections
*b* = 12.437 (2) Å	θ = 2.6–27.9°
*c* = 12.3864 (17) Å	µ = 0.95 mm^−^^1^
β = 102.028 (11)°	*T* = 150 K
*V* = 2061.1 (5) Å^3^	Block, purple
*Z* = 4	0.15 × 0.15 × 0.06 mm

### (II) Dichlorido(4,11-dimethyl-1,4,8,11-tetraazabicyclo[6.6.2]hexadecane)chromium(III) hexafluorophosphate Data collection

**Table:**

Stoe IPDS2 diffractometer	4158 independent reflections
Radiation source: fine-focus sealed tube	2073 reflections with *I* > 2σ(*I*)
Detector resolution: 6.67 pixels mm^-1^	*R*_int_ = 0.091
ω–scans	θ_max_ = 26.3°, θ_min_ = 2.6°
Absorption correction: analytical [a face-indexed absorption correction was applied; *X-AREA* (Stoe & Cie, 2002)]	*h* = −17→16
*T*_min_ = 0.778, *T*_max_ = 0.901	*k* = −15→13
13450 measured reflections	*l* = −15→15

### (II) Dichlorido(4,11-dimethyl-1,4,8,11-tetraazabicyclo[6.6.2]hexadecane)chromium(III) hexafluorophosphate Refinement

**Table:**

Refinement on *F*^2^	0 restraints
Least-squares matrix: full	Hydrogen site location: inferred from neighbouring sites
*R*[*F*^2^ > 2σ(*F*^2^)] = 0.045	H-atom parameters constrained
*wR*(*F*^2^) = 0.109	*w* = 1/[σ^2^(*F*_o_^2^) + (0.052*P*)^2^] where *P* = (*F*_o_^2^ + 2*F*_c_^2^)/3
*S* = 0.78	(Δ/σ)_max_ < 0.001
4158 reflections	Δρ_max_ = 0.33 e Å^−^^3^
255 parameters	Δρ_min_ = −0.79 e Å^−^^3^

### (II) Dichlorido(4,11-dimethyl-1,4,8,11-tetraazabicyclo[6.6.2]hexadecane)chromium(III) hexafluorophosphate Special details

**Table:**

Geometry. All e.s.d.'s (except the e.s.d. in the dihedral angle between two l.s. planes) are estimated using the full covariance matrix. The cell e.s.d.'s are taken into account individually in the estimation of e.s.d.'s in distances, angles and torsion angles; correlations between e.s.d.'s in cell parameters are only used when they are defined by crystal symmetry. An approximate (isotropic) treatment of cell e.s.d.'s is used for estimating e.s.d.'s involving l.s. planes.

### (II) Dichlorido(4,11-dimethyl-1,4,8,11-tetraazabicyclo[6.6.2]hexadecane)chromium(III) hexafluorophosphate Fractional atomic coordinates and isotropic or equivalent isotropic displacement parameters (Å^2^)

**Table:**

	*x*	*y*	*z*	*U*_iso_*/*U*_eq_	
Cr1	0.32108 (5)	0.56984 (5)	0.21705 (5)	0.03357 (19)	
Cl1	0.46100 (9)	0.62992 (9)	0.33888 (8)	0.0431 (3)	
Cl2	0.36519 (9)	0.66096 (9)	0.07251 (8)	0.0423 (3)	
N1	0.2651 (3)	0.4975 (3)	0.3442 (3)	0.0367 (8)	
N2	0.2251 (3)	0.7026 (3)	0.2372 (2)	0.0368 (9)	
N3	0.1984 (3)	0.4956 (3)	0.1138 (2)	0.0331 (8)	
N4	0.3972 (3)	0.4231 (3)	0.1961 (3)	0.0384 (9)	
C1	0.2305 (4)	0.5917 (3)	0.4026 (3)	0.0406 (11)	
H1A	0.2892	0.6311	0.4444	0.049*	
H1B	0.1910	0.5656	0.4558	0.049*	
C2	0.1674 (4)	0.6666 (4)	0.3209 (3)	0.0401 (11)	
H2A	0.1057	0.6291	0.2834	0.048*	
H2B	0.1478	0.7298	0.3600	0.048*	
C3	0.1559 (3)	0.7420 (4)	0.1358 (3)	0.0396 (10)	
H3A	0.1966	0.7733	0.0863	0.048*	
H3B	0.1151	0.8008	0.1573	0.048*	
C4	0.0858 (3)	0.6599 (4)	0.0706 (3)	0.0421 (11)	
H4A	0.0419	0.6316	0.1183	0.051*	
H4B	0.0425	0.6968	0.0074	0.051*	
C5	0.1360 (3)	0.5661 (4)	0.0269 (3)	0.0388 (10)	
H5A	0.0838	0.5211	−0.0193	0.047*	
H5B	0.1793	0.5943	−0.0214	0.047*	
C6	0.2446 (4)	0.4113 (4)	0.0536 (3)	0.0426 (11)	
H6A	0.2768	0.4460	−0.0020	0.051*	
H6B	0.1920	0.3623	0.0144	0.051*	
C7	0.3218 (4)	0.3474 (4)	0.1338 (3)	0.0416 (11)	
H7A	0.2891	0.3073	0.1856	0.050*	
H7B	0.3549	0.2949	0.0931	0.050*	
C8	0.4513 (3)	0.3706 (4)	0.3011 (3)	0.0408 (11)	
H8A	0.5060	0.4190	0.3371	0.049*	
H8B	0.4825	0.3035	0.2816	0.049*	
C9	0.3879 (4)	0.3433 (4)	0.3847 (3)	0.0421 (11)	
H9A	0.3353	0.2922	0.3496	0.051*	
H9B	0.4309	0.3055	0.4474	0.051*	
C10	0.3379 (3)	0.4366 (4)	0.4302 (3)	0.0405 (11)	
H10A	0.3021	0.4089	0.4862	0.049*	
H10B	0.3901	0.4869	0.4679	0.049*	
C11	0.1794 (3)	0.4237 (4)	0.3005 (3)	0.0395 (10)	
H11A	0.1286	0.4316	0.3463	0.047*	
H11B	0.2038	0.3485	0.3080	0.047*	
C12	0.1300 (3)	0.4441 (4)	0.1802 (3)	0.0402 (11)	
H12A	0.1056	0.3749	0.1454	0.048*	
H12B	0.0713	0.4913	0.1777	0.048*	
C13	0.2856 (4)	0.7989 (4)	0.2819 (3)	0.0443 (11)	
H13A	0.3262	0.7820	0.3548	0.053*	
H13B	0.2407	0.8589	0.2883	0.053*	
H13C	0.3294	0.8189	0.2318	0.053*	
C14	0.4760 (4)	0.4407 (4)	0.1303 (4)	0.0469 (12)	
H14A	0.5234	0.4952	0.1666	0.056*	
H14B	0.4447	0.4653	0.0560	0.056*	
H14C	0.5116	0.3731	0.1252	0.056*	
P1	0.12310 (10)	0.10057 (10)	0.18374 (9)	0.0430 (3)	
F1	0.0182 (2)	0.0745 (3)	0.1049 (2)	0.0696 (9)	
F2	0.0958 (3)	0.0212 (2)	0.2740 (2)	0.0688 (9)	
F3	0.2260 (2)	0.1297 (3)	0.2639 (2)	0.0718 (9)	
F4	0.1495 (2)	0.1810 (2)	0.0940 (2)	0.0637 (8)	
F5	0.1746 (2)	0.0057 (2)	0.1317 (2)	0.0615 (8)	
F6	0.0718 (2)	0.1974 (2)	0.2371 (2)	0.0664 (9)	

### (II) Dichlorido(4,11-dimethyl-1,4,8,11-tetraazabicyclo[6.6.2]hexadecane)chromium(III) hexafluorophosphate Atomic displacement parameters (Å^2^)

**Table:**

	*U*^11^	*U*^22^	*U*^33^	*U*^12^	*U*^13^	*U*^23^
Cr1	0.0378 (4)	0.0298 (4)	0.0323 (3)	−0.0011 (3)	0.0054 (3)	−0.0001 (3)
Cl1	0.0451 (7)	0.0383 (6)	0.0423 (5)	−0.0058 (5)	0.0008 (5)	0.0013 (4)
Cl2	0.0466 (7)	0.0428 (7)	0.0379 (5)	−0.0023 (5)	0.0096 (5)	0.0057 (4)
N1	0.040 (2)	0.0317 (19)	0.0366 (17)	0.0027 (17)	0.0037 (16)	0.0002 (14)
N2	0.045 (2)	0.033 (2)	0.0333 (16)	0.0000 (17)	0.0082 (16)	0.0005 (14)
N3	0.032 (2)	0.0315 (19)	0.0345 (16)	−0.0029 (16)	0.0053 (15)	0.0008 (14)
N4	0.043 (2)	0.035 (2)	0.0374 (17)	0.0034 (18)	0.0077 (16)	0.0012 (15)
C1	0.052 (3)	0.035 (3)	0.035 (2)	0.002 (2)	0.011 (2)	−0.0052 (17)
C2	0.047 (3)	0.031 (2)	0.044 (2)	0.003 (2)	0.013 (2)	−0.0005 (18)
C3	0.040 (3)	0.036 (3)	0.040 (2)	0.004 (2)	0.0009 (19)	0.0013 (18)
C4	0.042 (3)	0.040 (3)	0.042 (2)	0.004 (2)	0.003 (2)	0.004 (2)
C5	0.039 (3)	0.037 (3)	0.037 (2)	0.001 (2)	0.0000 (19)	0.0011 (18)
C6	0.050 (3)	0.037 (3)	0.041 (2)	0.002 (2)	0.008 (2)	−0.0053 (19)
C7	0.052 (3)	0.034 (2)	0.040 (2)	0.003 (2)	0.011 (2)	−0.0031 (19)
C8	0.043 (3)	0.036 (3)	0.040 (2)	0.010 (2)	0.003 (2)	0.0025 (18)
C9	0.044 (3)	0.040 (3)	0.040 (2)	0.004 (2)	0.005 (2)	0.0066 (19)
C10	0.045 (3)	0.038 (3)	0.035 (2)	0.001 (2)	0.002 (2)	0.0054 (18)
C11	0.043 (3)	0.038 (2)	0.037 (2)	−0.005 (2)	0.007 (2)	0.0022 (19)
C12	0.047 (3)	0.035 (3)	0.039 (2)	−0.009 (2)	0.009 (2)	0.0011 (18)
C13	0.051 (3)	0.031 (3)	0.049 (2)	−0.004 (2)	0.007 (2)	−0.0061 (19)
C14	0.042 (3)	0.050 (3)	0.050 (2)	0.004 (2)	0.014 (2)	0.005 (2)
P1	0.0498 (8)	0.0337 (7)	0.0480 (6)	−0.0055 (6)	0.0157 (6)	−0.0035 (5)
F1	0.057 (2)	0.078 (2)	0.0717 (18)	−0.0152 (17)	0.0075 (15)	−0.0207 (16)
F2	0.097 (3)	0.057 (2)	0.0623 (16)	−0.0099 (18)	0.0399 (17)	0.0102 (14)
F3	0.061 (2)	0.088 (3)	0.0633 (17)	−0.0109 (18)	0.0054 (15)	−0.0088 (16)
F4	0.079 (2)	0.0471 (18)	0.0706 (17)	−0.0034 (16)	0.0279 (16)	0.0136 (14)
F5	0.084 (2)	0.0391 (16)	0.0712 (17)	0.0070 (16)	0.0400 (16)	−0.0038 (13)
F6	0.069 (2)	0.0465 (18)	0.092 (2)	−0.0049 (16)	0.0352 (17)	−0.0261 (15)

### (II) Dichlorido(4,11-dimethyl-1,4,8,11-tetraazabicyclo[6.6.2]hexadecane)chromium(III) hexafluorophosphate Geometric parameters (Å, º)

**Table:**

Cr1—N1	2.093 (3)	C6—C7	1.516 (6)
Cr1—N3	2.100 (3)	C6—H6A	0.9900
Cr1—N4	2.143 (4)	C6—H6B	0.9900
Cr1—N2	2.155 (4)	C7—H7A	0.9900
Cr1—Cl1	2.3012 (13)	C7—H7B	0.9900
Cr1—Cl2	2.3031 (12)	C8—C9	1.520 (6)
N1—C11	1.499 (5)	C8—H8A	0.9900
N1—C10	1.502 (5)	C8—H8B	0.9900
N1—C1	1.504 (5)	C9—C10	1.516 (6)
N2—C3	1.490 (5)	C9—H9A	0.9900
N2—C13	1.495 (5)	C9—H9B	0.9900
N2—C2	1.497 (5)	C10—H10A	0.9900
N3—C6	1.500 (5)	C10—H10B	0.9900
N3—C5	1.507 (5)	C11—C12	1.524 (5)
N3—C12	1.511 (5)	C11—H11A	0.9900
N4—C7	1.487 (6)	C11—H11B	0.9900
N4—C14	1.498 (5)	C12—H12A	0.9900
N4—C8	1.505 (5)	C12—H12B	0.9900
C1—C2	1.508 (6)	C13—H13A	0.9800
C1—H1A	0.9900	C13—H13B	0.9800
C1—H1B	0.9900	C13—H13C	0.9800
C2—H2A	0.9900	C14—H14A	0.9800
C2—H2B	0.9900	C14—H14B	0.9800
C3—C4	1.514 (6)	C14—H14C	0.9800
C3—H3A	0.9900	P1—F5	1.579 (3)
C3—H3B	0.9900	P1—F3	1.587 (3)
C4—C5	1.510 (6)	P1—F1	1.592 (3)
C4—H4A	0.9900	P1—F4	1.592 (3)
C4—H4B	0.9900	P1—F2	1.593 (3)
C5—H5A	0.9900	P1—F6	1.605 (3)
C5—H5B	0.9900		
			
N1—Cr1—N3	84.18 (13)	N3—C6—C7	110.4 (3)
N1—Cr1—N4	89.33 (13)	N3—C6—H6A	109.6
N3—Cr1—N4	84.16 (14)	C7—C6—H6A	109.6
N1—Cr1—N2	85.11 (13)	N3—C6—H6B	109.6
N3—Cr1—N2	88.80 (14)	C7—C6—H6B	109.6
N4—Cr1—N2	171.44 (14)	H6A—C6—H6B	108.1
N1—Cr1—Cl1	91.75 (10)	N4—C7—C6	108.7 (4)
N3—Cr1—Cl1	172.78 (10)	N4—C7—H7A	109.9
N4—Cr1—Cl1	89.84 (10)	C6—C7—H7A	109.9
N2—Cr1—Cl1	96.82 (10)	N4—C7—H7B	109.9
N1—Cr1—Cl2	173.17 (11)	C6—C7—H7B	109.9
N3—Cr1—Cl2	92.77 (9)	H7A—C7—H7B	108.3
N4—Cr1—Cl2	96.45 (9)	N4—C8—C9	115.9 (4)
N2—Cr1—Cl2	88.73 (9)	N4—C8—H8A	108.3
Cl1—Cr1—Cl2	91.90 (5)	C9—C8—H8A	108.3
C11—N1—C10	107.4 (3)	N4—C8—H8B	108.3
C11—N1—C1	110.5 (3)	C9—C8—H8B	108.3
C10—N1—C1	106.3 (3)	H8A—C8—H8B	107.4
C11—N1—Cr1	111.9 (2)	C10—C9—C8	116.6 (4)
C10—N1—Cr1	117.3 (3)	C10—C9—H9A	108.1
C1—N1—Cr1	103.2 (2)	C8—C9—H9A	108.1
C3—N2—C13	104.7 (3)	C10—C9—H9B	108.1
C3—N2—C2	110.4 (3)	C8—C9—H9B	108.1
C13—N2—C2	108.3 (3)	H9A—C9—H9B	107.3
C3—N2—Cr1	116.7 (2)	N1—C10—C9	114.0 (3)
C13—N2—Cr1	110.7 (3)	N1—C10—H10A	108.8
C2—N2—Cr1	105.9 (2)	C9—C10—H10A	108.8
C6—N3—C5	106.6 (3)	N1—C10—H10B	108.8
C6—N3—C12	110.3 (3)	C9—C10—H10B	108.8
C5—N3—C12	108.2 (3)	H10A—C10—H10B	107.7
C6—N3—Cr1	104.1 (3)	N1—C11—C12	113.9 (3)
C5—N3—Cr1	116.2 (3)	N1—C11—H11A	108.8
C12—N3—Cr1	111.2 (2)	C12—C11—H11A	108.8
C7—N4—C14	108.1 (3)	N1—C11—H11B	108.8
C7—N4—C8	109.7 (3)	C12—C11—H11B	108.8
C14—N4—C8	104.7 (3)	H11A—C11—H11B	107.7
C7—N4—Cr1	107.5 (3)	N3—C12—C11	113.9 (4)
C14—N4—Cr1	111.4 (3)	N3—C12—H12A	108.8
C8—N4—Cr1	115.4 (2)	C11—C12—H12A	108.8
N1—C1—C2	110.6 (3)	N3—C12—H12B	108.8
N1—C1—H1A	109.5	C11—C12—H12B	108.8
C2—C1—H1A	109.5	H12A—C12—H12B	107.7
N1—C1—H1B	109.5	N2—C13—H13A	109.5
C2—C1—H1B	109.5	N2—C13—H13B	109.5
H1A—C1—H1B	108.1	H13A—C13—H13B	109.5
N2—C2—C1	109.8 (4)	N2—C13—H13C	109.5
N2—C2—H2A	109.7	H13A—C13—H13C	109.5
C1—C2—H2A	109.7	H13B—C13—H13C	109.5
N2—C2—H2B	109.7	N4—C14—H14A	109.5
C1—C2—H2B	109.7	N4—C14—H14B	109.5
H2A—C2—H2B	108.2	H14A—C14—H14B	109.5
N2—C3—C4	116.7 (4)	N4—C14—H14C	109.5
N2—C3—H3A	108.1	H14A—C14—H14C	109.5
C4—C3—H3A	108.1	H14B—C14—H14C	109.5
N2—C3—H3B	108.1	F5—P1—F3	90.73 (18)
C4—C3—H3B	108.1	F5—P1—F1	91.12 (17)
H3A—C3—H3B	107.3	F3—P1—F1	178.15 (19)
C5—C4—C3	115.3 (4)	F5—P1—F4	89.96 (15)
C5—C4—H4A	108.5	F3—P1—F4	89.57 (17)
C3—C4—H4A	108.5	F1—P1—F4	90.36 (17)
C5—C4—H4B	108.5	F5—P1—F2	90.75 (16)
C3—C4—H4B	108.5	F3—P1—F2	90.55 (17)
H4A—C4—H4B	107.5	F1—P1—F2	89.50 (18)
N3—C5—C4	115.2 (3)	F4—P1—F2	179.28 (19)
N3—C5—H5A	108.5	F5—P1—F6	179.47 (19)
C4—C5—H5A	108.5	F3—P1—F6	88.75 (17)
N3—C5—H5B	108.5	F1—P1—F6	89.40 (17)
C4—C5—H5B	108.5	F4—P1—F6	89.91 (17)
H5A—C5—H5B	107.5	F2—P1—F6	89.38 (16)
			
C11—N1—C1—C2	−71.2 (4)	C8—N4—C7—C6	159.7 (3)
C10—N1—C1—C2	172.6 (4)	Cr1—N4—C7—C6	33.5 (4)
Cr1—N1—C1—C2	48.6 (4)	N3—C6—C7—N4	−56.2 (5)
C3—N2—C2—C1	159.5 (3)	C7—N4—C8—C9	−64.3 (5)
C13—N2—C2—C1	−86.4 (4)	C14—N4—C8—C9	−180.0 (4)
Cr1—N2—C2—C1	32.3 (4)	Cr1—N4—C8—C9	57.2 (4)
N1—C1—C2—N2	−56.7 (5)	N4—C8—C9—C10	−61.3 (5)
C13—N2—C3—C4	178.8 (4)	C11—N1—C10—C9	66.6 (5)
C2—N2—C3—C4	−64.9 (5)	C1—N1—C10—C9	−175.1 (4)
Cr1—N2—C3—C4	56.0 (5)	Cr1—N1—C10—C9	−60.3 (4)
N2—C3—C4—C5	−60.3 (5)	C8—C9—C10—N1	61.5 (5)
C6—N3—C5—C4	−177.6 (4)	C10—N1—C11—C12	−149.2 (4)
C12—N3—C5—C4	63.7 (5)	C1—N1—C11—C12	95.2 (4)
Cr1—N3—C5—C4	−62.1 (4)	Cr1—N1—C11—C12	−19.2 (5)
C3—C4—C5—N3	62.9 (5)	C6—N3—C12—C11	95.5 (4)
C5—N3—C6—C7	170.9 (4)	C5—N3—C12—C11	−148.2 (4)
C12—N3—C6—C7	−71.8 (4)	Cr1—N3—C12—C11	−19.6 (4)
Cr1—N3—C6—C7	47.6 (4)	N1—C11—C12—N3	25.8 (5)
C14—N4—C7—C6	−86.8 (4)		

### (II) Dichlorido(4,11-dimethyl-1,4,8,11-tetraazabicyclo[6.6.2]hexadecane)chromium(III) hexafluorophosphate Hydrogen-bond geometry (Å, º)

**Table:**

*D*—H···*A*	*D*—H	H···*A*	*D*···*A*	*D*—H···*A*
C1—H1*B*···F5^i^	0.99	2.41	3.318 (5)	153
C2—H2*A*···F1^ii^	0.99	2.50	3.094 (5)	119
C2—H2*A*···F6^ii^	0.99	2.53	3.223 (6)	127
C7—H7*A*···F4	0.99	2.55	3.098 (6)	115
C12—H12*B*···F2^ii^	0.99	2.51	3.393 (6)	149
C3—H3*A*···Cl2	0.99	2.73	3.280 (5)	115
C5—H5*B*···Cl2	0.99	2.70	3.287 (5)	119
C8—H8*A*···Cl1	0.99	2.70	3.258 (5)	116
C8—H8*B*···Cl1^iii^	0.99	2.82	3.778 (4)	162
C10—H10*B*···Cl1	0.99	2.70	3.270 (5)	117
C13—H13*A*···Cl1	0.98	2.68	3.157 (5)	111
C13—H13*A*···Cl2^iv^	0.98	2.73	3.572 (4)	144
C14—H14*B*···Cl2	0.98	2.69	3.141 (5)	108

Symmetry codes: (i) *x*, −*y*+1/2, *z*+1/2; (ii) −*x*, *y*+1/2, −*z*+1/2; (iii) −*x*+1, *y*−1/2, −*z*+1/2; (iv) *x*, −*y*+3/2, *z*+1/2.

## References

[bb1] Boswell, C. A., Sun, X., Niu, W., Weisman, G. R., Wong, E. H., Rheingold, A. L. & Anderson, C. J. (2004). *J. Med. Chem.* **47**, 1465–1474. 10.1021/jm030383m 14998334

[bb2] Cotton, F. A. & Wilkinson, G. (1988). In *Advanced Inorganic Chemistry*, 5th ed. New York: Wiley.

[bb3] Dong, L., Wang, Y., Lu, Y., Chen, Z., Mei, F., Xiong, H. & Yin, G. (2013). *Inorg. Chem.* **52**, 5418–5427. 10.1021/ic400361s 23600453

[bb4] Hubin, T. J. (2003). *Coord. Chem. Rev.* **241**, 27–46.

[bb5] Hubin, T. J., Alcock, N. W., Clase, H. J., Seib, L. L. & Busch, D. H. (2002). *Inorg. Chim. Acta*, **337**, 91–102.

[bb6] Hubin, T. J., McCormick, J. M., Alcock, N. W. & Busch, D. H. (2001). *Inorg. Chem.* **40**, 435–444. 10.1021/ic9912225 11209599

[bb7] Lewis, E. A., Hubin, T. J. & Archibald, S. J. (2005). Patent WO2005121109.

[bb8] Maples, D. L., Maples, R. D., Hoffert, W. A., Parsell, T. H., van Asselt, A., Silversides, J. D., Archibald, S. J. & Hubin, T. J. (2009). *Inorg. Chim. Acta*, **362**, 2084–2088. 10.1016/j.ica.2008.09.034 PMC2746693 20161052

[bb9] McClain II, J. M., Maples, D. L., Maples, R. D., Matz, D. L., Harris, S. M., Nelson, A. D. L., Silversides, J. D., Archibald, S. J. & Hubin, T. J. (2006). *Acta Cryst.* C**62**, m553–m555. 10.1107/S0108270106040765 17088625

[bb10] Orpen, G. A., Brammer, L., Allen, F. H., Kennard, O., Watson, D. G. & Taylor, R. J. (1989). *J. Chem. Soc. Dalton Trans.* pp. S1–S83.

[bb11] Sheldrick, G. M. (2008). *Acta Cryst.* A**64**, 112–122. 10.1107/S0108767307043930 18156677

[bb12] Silversides, J. D., Smith, R. & Archibald, S. J. (2011). *Dalton Trans.* **40**, 6289–6297. 10.1039/c0dt01395a 21455520

[bb13] Smith, R., Huskens, D., Daelemans, D., Mewis, R. E., Garcia, C. D., Cain, A. N., Carder Freeman, T. N., Pannecouque, C., De Clercq, E., Schols, D., Hubin, T. J. & Archibald, S. J. (2012). *Dalton Trans.* **41**, 11369–11377. 10.1039/c2dt31137b 22892890

[bb14] Sprague, J. E., Peng, Y., Fiamengo, A. L., Woodin, K. S., Southwick, E. A., Weisman, G. R., Wong, E. H., Golen, J. A., Rheingold, A. L. & Anderson, C. J. (2007). *J. Med. Chem.* **50**, 2527–2535. 10.1021/jm070204r 17458949

[bb15] Stoe & Cie (2002). *X-AREA* and *X-RED*. Stoe & Cie GmbH, Darmstadt, Germany.

[bb16] Valks, G. C., McRobbie, G., Lewis, E. A., Hubin, T. J., Hunter, T. M., Sadler, P. J., Pannecouque, C., De Clerq, E. & Archibald, S. J. (2006). *J. Med. Chem.* **49**, 6162–6165. 10.1021/jm0607810 17034122

[bb17] Weisman, G. R., Rogers, M. E., Wong, E. H., Jasinski, J. P. & Paight, E. S. (1990). *J. Am. Chem. Soc.* **112**, 8604–8605.

[bb18] Wong, E. H., Weisman, G. R., Hill, D. C., Reed, D. P., Rogers, M. E., Condon, J. S., Fagan, M. A., Calabrese, J. C., Lam, K.-C., Guzei, I. A. & Rheingold, A. L. (2000). *J. Am. Chem. Soc.* **122**, 10561–10572.

[bb19] Yin, G., Danby, A. M., Kitko, D., Carter, J. D., Scheper, W. M. & Busch, D. H. (2007). *J. Am. Chem. Soc.* **129**, 1512–1513. 10.1021/ja0673229 17249671

